# 1,1′-Dimethyl-4,4′-(propane-1,3-di­yl)dipyridinium tetra­bromidocadmate(II)

**DOI:** 10.1107/S1600536808030092

**Published:** 2008-09-24

**Authors:** Fei-Fei Li, Zhi-Gang Li, Jian-Cheng Deng, Jing-Wei Xu

**Affiliations:** a The College of Chemistry, Xiangtan University, Hunan 411105, People’s Republic of China; b National Analytical Research Center of Electrochemistry and Spectroscopy, Changchun Institute of Applied Chemistry, Chinese Academy of Sciences, Changchun 130022, People’s Republic of China; c Graduate School of Chinese Academy of Sciences, Beijing 100039, People’s Republic of China

## Abstract

In the cation of the title compound, (C_15_H_20_N_2_)[CdBr_4_], the dihedral angle between the two pyridine rings is 70.85 (5)°. An inter­molecular π–π inter­action between the pyridine rings [centroid–centroid distance = 3.900 (4) Å] is observed. The Cd^II^ atom has a distorted tetra­hedral coordination.

## Related literature

For related structures, see: Dou *et al.* (2007 ▶); Yang *et al.* (2008 ▶).
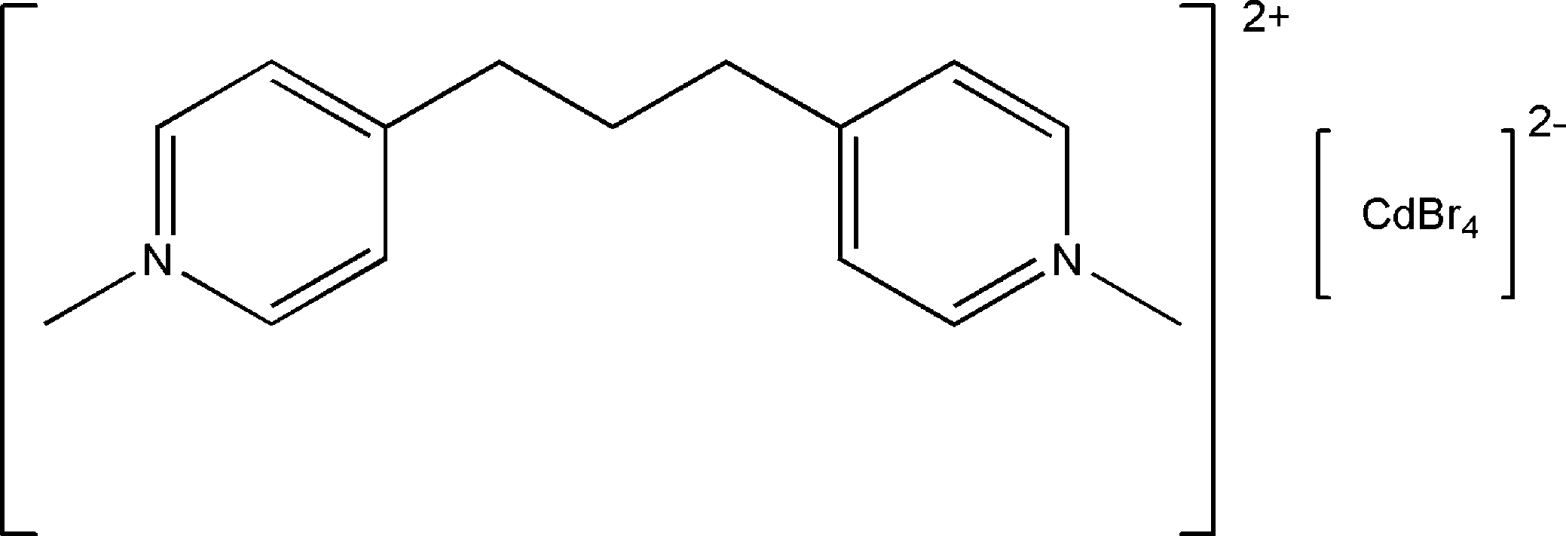


## Experimental

### 

#### Crystal data

(C_15_H_20_N_2_)[CdBr_4_]
*M* *_r_* = 660.37
Monoclinic, 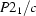

*a* = 15.422 (2) Å
*b* = 15.382 (2) Å
*c* = 8.9885 (14) Å
β = 105.171 (3)°
*V* = 2058.0 (5) Å^3^
*Z* = 4
Mo *K*α radiation
μ = 8.82 mm^−1^
*T* = 293 (2) K
0.28 × 0.19 × 0.11 mm

#### Data collection

Bruker APEX CCD area-detector diffractometer
Absorption correction: multi-scan (**SADABS**; Sheldrick, 2003 ▶) *T*_min_ = 0.148, *T*_max_ = 0.379
11428 measured reflections
4042 independent reflections
2181 reflections with *I* > 2σ(*I*)
*R*_int_ = 0.066

#### Refinement

*R*[*F* ^2^ > 2σ(*F* ^2^)] = 0.053
*wR*(*F* ^2^) = 0.112
*S* = 1.01
4042 reflections
201 parameters
H-atom parameters constrained
Δρ_max_ = 1.23 e Å^−3^
Δρ_min_ = −0.42 e Å^−3^

### 

Data collection: *SMART* (Bruker, 1998 ▶); cell refinement: *SAINT-Plus* (Bruker, 2003 ▶); data reduction: *SAINT-Plus*; program(s) used to solve structure: *SHELXS97* (Sheldrick, 2008 ▶); program(s) used to refine structure: *SHELXL97* (Sheldrick, 2008 ▶); molecular graphics: *SHELXTL* (Sheldrick, 2008 ▶); software used to prepare material for publication: *SHELXTL*.

## Supplementary Material

Crystal structure: contains datablocks global, I. DOI: 10.1107/S1600536808030092/is2330sup1.cif

Structure factors: contains datablocks I. DOI: 10.1107/S1600536808030092/is2330Isup2.hkl

Additional supplementary materials:  crystallographic information; 3D view; checkCIF report

## Figures and Tables

**Fig. 1.**
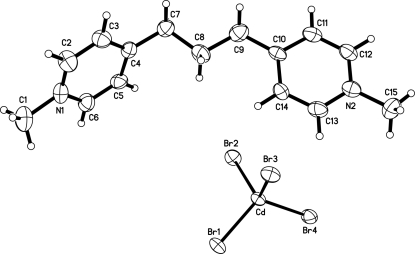
The molecular structure of (I), with the atom-labeling scheme and 30% probability displacement ellipsoids.

**Fig. 2.**
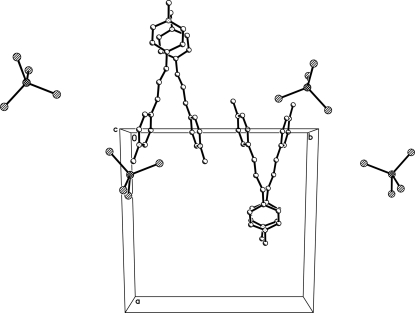
A partial packing view of (I) along the c axis. For the sake of clarity, H atoms have been omitted.

## supplementary crystallographic information

### Comment

Dou *et al.* (2007) and Yang *et al.* (2008) have reported crystal structures of two related compounds synthesized by *in situ* reaction under hydrothermal condition, in which pyridine N atoms were covalently bonded to methyl groups and the counterions were ClO_4_^-^ and BF_4_^-^ anions. Here, we report the structure of the title compound, (I), synthesized by the same method, in which the counter-ion is tetrabromocadmate(II).

Compound (I), as shown in Fig. 1, consists of a 1,3-bis(1-methyl-4-pyridinium)propane cation and a tetrabormocadmate anion. As result of the flexible propane chain, the two pyridine rings have seriously torsion with the dihedral angle of 70.85 (5)°. The Cd^II^ atom is coordinated by four Br atoms to a tetrahedral divalent anion.

The crystal structure is stabilized by a weak π–π stacking interaction between adjacent pyridine rings, the shortest atom-to-atom^i^, centroid-centroid^i^ and interplanar distances being 3.678 (4), 3.900 (4) and 3.638 (3) Å, respectively [symmetry code: (i) -*x*, 1 - *y*, 1 - *z*]. The pyridine rings also contact to each other, the shortest atom-to-atom^ii^ being 3.621 (3) Å [symmetry code: (ii) *x*, 3/2 - *y*, -1/2 + *z*], which leads to a supramolecular chain (Fig. 2).

### Experimental

Compound (I) was solvothermally prepared from a reaction mixture of CdBr_2_ (0.2 mmol), 1,3-bis(4-pyridyl)propane (0.1 mmol), methanol (3 ml) and distilled water (8 ml); the pH value was adjusted to 4.6 with trimethylamine and acetic acid. The mixture was stirred for 20 min at room temperature and then sealed in a Teflon-lined stainless steel autoclave with a 23 ml capacity at 428 K for 72 h. After cooling to room temperature, the filtered solution was slowly evaporates and 7 days later colourless block-shaped crystals were obtained; these were washed with deionized water, filtered, and dried in air (yield 46% based on Cd).

### Refinement

H atoms were placed geometrically (C—H = 0.93–0.97 Å) and refined as riding, with *U*_iso_(H) = 1.2*U*_eq_(C) or 1.5*U*_eq_(methyl C) . The highest residual electron density peak is located at 1.223 (3) Å from Cd atom.

### Crystal data

**Table:**

(C_15_H_20_N_2_)[CdBr_4_]	*F*(000) = 1248
*M**_r_* = 660.37	*D*_x_ = 2.131 Mg m^−^^3^
Monoclinic, *P*2_1_/*c*	Mo *K*α radiation, λ = 0.71073 Å
Hall symbol: -P 2ybc	Cell parameters from 1386 reflections
*a* = 15.422 (2) Å	θ = 2.7–19.5°
*b* = 15.382 (2) Å	µ = 8.83 mm^−^^1^
*c* = 8.9885 (14) Å	*T* = 293 K
β = 105.171 (3)°	Block, white
*V* = 2058.0 (5) Å^3^	0.28 × 0.19 × 0.11 mm
*Z* = 4	

### Data collection

**Table:**

Bruker APEX CCD area-detector diffractometer	4042 independent reflections
Radiation source: fine-focus sealed tube	2181 reflections with *I* > 2σ(*I*)
graphite	*R*_int_ = 0.066
φ and ω scans	θ_max_ = 26.1°, θ_min_ = 1.9°
Absorption correction: multi-scan (*SADABS*; Sheldrick, 2003)	*h* = −19→18
*T*_min_ = 0.148, *T*_max_ = 0.379	*k* = −19→18
11428 measured reflections	*l* = −11→5

### Refinement

**Table:**

Refinement on *F*^2^	Primary atom site location: structure-invariant direct methods
Least-squares matrix: full	Secondary atom site location: difference Fourier map
*R*[*F*^2^ > 2σ(*F*^2^)] = 0.053	Hydrogen site location: inferred from neighbouring sites
*wR*(*F*^2^) = 0.112	H-atom parameters constrained
*S* = 1.01	*w* = 1/[σ^2^(*F*_o_^2^) + (0.0341*P*)^2^ + 0.3024*P*] where *P* = (*F*_o_^2^ + 2*F*_c_^2^)/3
4042 reflections	(Δ/σ)_max_ = 0.003
201 parameters	Δρ_max_ = 1.23 e Å^−^^3^
0 restraints	Δρ_min_ = −0.42 e Å^−^^3^

### Special details

**Table:**

Geometry. All e.s.d.'s (except the e.s.d. in the dihedral angle between two l.s. planes) are estimated using the full covariance matrix. The cell e.s.d.'s are taken into account individually in the estimation of e.s.d.'s in distances, angles and torsion angles; correlations between e.s.d.'s in cell parameters are only used when they are defined by crystal symmetry. An approximate (isotropic) treatment of cell e.s.d.'s is used for estimating e.s.d.'s involving l.s. planes.
Refinement. Refinement of *F*^2^ against ALL reflections. The weighted *R*-factor *wR* and goodness of fit *S* are based on *F*^2^, conventional *R*-factors *R* are based on *F*, with *F* set to zero for negative *F*^2^. The threshold expression of *F*^2^ > σ(*F*^2^) is used only for calculating *R*-factors(gt) *etc*. and is not relevant to the choice of reflections for refinement. *R*-factors based on *F*^2^ are statistically about twice as large as those based on *F*, and *R*- factors based on ALL data will be even larger.

### Fractional atomic coordinates and isotropic or equivalent isotropic displacement parameters (Å^2^)

**Table:**

	*x*	*y*	*z*	*U*_iso_*/*U*_eq_	
Cd	0.24235 (4)	0.02630 (4)	0.65224 (8)	0.0576 (2)	
Br1	0.36144 (6)	0.00463 (5)	0.49814 (12)	0.0704 (3)	
Br2	0.32230 (7)	0.00571 (6)	0.94342 (11)	0.0768 (3)	
Br3	0.17908 (7)	0.18161 (6)	0.59855 (12)	0.0768 (3)	
Br4	0.11503 (7)	−0.08446 (7)	0.58003 (13)	0.0872 (4)	
N1	0.5535 (4)	0.7487 (5)	0.6414 (8)	0.0596 (19)	
N2	−0.0832 (5)	0.6007 (4)	0.4141 (8)	0.0561 (18)	
C1	0.6335 (6)	0.7530 (6)	0.5741 (13)	0.090 (3)	
H1A	0.6335	0.8077	0.5227	0.135*	
H1B	0.6301	0.7065	0.5016	0.135*	
H1C	0.6878	0.7475	0.6553	0.135*	
C2	0.5137 (6)	0.8200 (5)	0.6755 (11)	0.069 (3)	
H2	0.5361	0.8743	0.6591	0.083*	
C3	0.4410 (6)	0.8150 (5)	0.7338 (10)	0.064 (2)	
H3	0.4151	0.8658	0.7584	0.077*	
C4	0.4045 (5)	0.7344 (5)	0.7575 (9)	0.051 (2)	
C5	0.4487 (6)	0.6626 (5)	0.7242 (9)	0.058 (2)	
H5	0.4286	0.6075	0.7420	0.070*	
C6	0.5217 (5)	0.6701 (5)	0.6652 (10)	0.064 (2)	
H6	0.5496	0.6203	0.6414	0.077*	
C7	0.3210 (5)	0.7281 (5)	0.8100 (10)	0.061 (2)	
H7A	0.3059	0.7850	0.8426	0.073*	
H7B	0.3309	0.6891	0.8977	0.073*	
C8	0.2434 (5)	0.6944 (5)	0.6807 (10)	0.063 (2)	
H8A	0.2598	0.6388	0.6449	0.076*	
H8B	0.2319	0.7348	0.5949	0.076*	
C9	0.1586 (6)	0.6836 (6)	0.7355 (10)	0.070 (3)	
H9A	0.1716	0.6427	0.8206	0.083*	
H9B	0.1453	0.7391	0.7757	0.083*	
C10	0.0753 (5)	0.6531 (5)	0.6192 (10)	0.048 (2)	
C11	−0.0041 (6)	0.6486 (5)	0.6605 (11)	0.064 (2)	
H11	−0.0055	0.6634	0.7601	0.076*	
C12	−0.0813 (6)	0.6226 (5)	0.5567 (11)	0.063 (2)	
H12	−0.1342	0.6202	0.5877	0.075*	
C13	−0.0069 (6)	0.6036 (5)	0.3678 (10)	0.062 (2)	
H13	−0.0074	0.5884	0.2674	0.075*	
C14	0.0719 (6)	0.6293 (5)	0.4708 (11)	0.061 (2)	
H14	0.1245	0.6305	0.4385	0.073*	
C15	−0.1667 (6)	0.5751 (6)	0.3004 (11)	0.078 (3)	
H15A	−0.2122	0.5625	0.3526	0.117*	
H15B	−0.1558	0.5244	0.2457	0.117*	
H15C	−0.1867	0.6219	0.2288	0.117*	

### Atomic displacement parameters (Å^2^)

**Table:**

	*U*^11^	*U*^22^	*U*^33^	*U*^12^	*U*^13^	*U*^23^
Cd	0.0653 (4)	0.0532 (4)	0.0608 (4)	0.0054 (3)	0.0283 (3)	0.0055 (3)
Br1	0.0749 (6)	0.0653 (5)	0.0857 (7)	0.0108 (4)	0.0469 (6)	0.0137 (5)
Br2	0.0856 (7)	0.0841 (6)	0.0634 (7)	0.0073 (5)	0.0244 (6)	0.0206 (5)
Br3	0.0986 (7)	0.0574 (5)	0.0807 (7)	0.0206 (5)	0.0345 (6)	0.0085 (5)
Br4	0.0920 (7)	0.0907 (7)	0.0933 (8)	−0.0277 (6)	0.0496 (7)	−0.0219 (6)
N1	0.048 (4)	0.063 (5)	0.064 (5)	−0.004 (4)	0.008 (4)	0.015 (4)
N2	0.064 (5)	0.054 (4)	0.054 (5)	0.001 (3)	0.022 (4)	0.004 (4)
C1	0.072 (6)	0.083 (7)	0.117 (9)	−0.013 (5)	0.027 (7)	0.013 (6)
C2	0.075 (6)	0.047 (5)	0.089 (8)	0.004 (5)	0.029 (6)	0.018 (5)
C3	0.074 (6)	0.054 (5)	0.067 (7)	0.005 (5)	0.023 (5)	0.001 (5)
C4	0.055 (5)	0.051 (5)	0.042 (5)	−0.005 (4)	0.008 (4)	0.004 (4)
C5	0.076 (6)	0.040 (5)	0.064 (6)	−0.013 (4)	0.027 (5)	−0.009 (4)
C6	0.065 (6)	0.055 (5)	0.075 (7)	−0.007 (4)	0.020 (5)	0.000 (5)
C7	0.067 (6)	0.058 (5)	0.062 (6)	−0.003 (4)	0.022 (5)	0.002 (5)
C8	0.071 (6)	0.063 (5)	0.062 (7)	−0.006 (5)	0.029 (5)	0.005 (5)
C9	0.079 (6)	0.076 (6)	0.056 (6)	−0.009 (5)	0.021 (6)	0.001 (5)
C10	0.059 (5)	0.047 (4)	0.045 (5)	0.003 (4)	0.028 (5)	0.008 (4)
C11	0.069 (6)	0.073 (6)	0.055 (6)	0.008 (5)	0.026 (6)	−0.006 (5)
C12	0.064 (6)	0.075 (6)	0.058 (7)	0.011 (5)	0.032 (6)	0.003 (5)
C13	0.074 (6)	0.069 (5)	0.052 (6)	−0.006 (5)	0.031 (6)	0.000 (5)
C14	0.053 (6)	0.074 (6)	0.067 (7)	−0.008 (4)	0.033 (5)	−0.006 (5)
C15	0.075 (6)	0.086 (7)	0.068 (7)	−0.006 (5)	0.009 (6)	0.006 (6)

### Geometric parameters (Å, °)

**Table:**

Cd—Br4	2.5520 (11)	C6—H6	0.9300
Cd—Br3	2.5779 (11)	C7—C8	1.524 (10)
Cd—Br1	2.5951 (11)	C7—H7A	0.9700
Cd—Br2	2.6041 (12)	C7—H7B	0.9700
N1—C2	1.332 (10)	C8—C9	1.522 (10)
N1—C6	1.342 (9)	C8—H8A	0.9700
N1—C1	1.512 (11)	C8—H8B	0.9700
N2—C12	1.318 (10)	C9—C10	1.503 (11)
N2—C13	1.347 (10)	C9—H9A	0.9700
N2—C15	1.473 (10)	C9—H9B	0.9700
C1—H1A	0.9600	C10—C14	1.370 (10)
C1—H1B	0.9600	C10—C11	1.373 (10)
C1—H1C	0.9600	C11—C12	1.365 (11)
C2—C3	1.359 (11)	C11—H11	0.9300
C2—H2	0.9300	C12—H12	0.9300
C3—C4	1.401 (10)	C13—C14	1.380 (11)
C3—H3	0.9300	C13—H13	0.9300
C4—C5	1.371 (10)	C14—H14	0.9300
C4—C7	1.486 (10)	C15—H15A	0.9600
C5—C6	1.370 (11)	C15—H15B	0.9600
C5—H5	0.9300	C15—H15C	0.9600
			
Br4—Cd—Br3	110.04 (4)	C4—C7—H7B	109.5
Br4—Cd—Br1	112.55 (4)	C8—C7—H7B	109.5
Br3—Cd—Br1	107.77 (4)	H7A—C7—H7B	108.1
Br4—Cd—Br2	107.75 (4)	C7—C8—C9	111.1 (7)
Br3—Cd—Br2	110.93 (4)	C7—C8—H8A	109.4
Br1—Cd—Br2	107.80 (4)	C9—C8—H8A	109.4
C2—N1—C6	119.8 (8)	C7—C8—H8B	109.4
C2—N1—C1	122.0 (7)	C9—C8—H8B	109.4
C6—N1—C1	118.3 (8)	H8A—C8—H8B	108.0
C12—N2—C13	119.6 (8)	C10—C9—C8	117.3 (7)
C12—N2—C15	122.4 (8)	C10—C9—H9A	108.0
C13—N2—C15	118.0 (8)	C8—C9—H9A	108.0
N1—C1—H1A	109.5	C10—C9—H9B	108.0
N1—C1—H1B	109.5	C8—C9—H9B	108.0
H1A—C1—H1B	109.5	H9A—C9—H9B	107.2
N1—C1—H1C	109.5	C14—C10—C11	116.1 (8)
H1A—C1—H1C	109.5	C14—C10—C9	124.7 (8)
H1B—C1—H1C	109.5	C11—C10—C9	119.2 (8)
N1—C2—C3	121.2 (8)	C12—C11—C10	120.7 (8)
N1—C2—H2	119.4	C12—C11—H11	119.7
C3—C2—H2	119.4	C10—C11—H11	119.7
C2—C3—C4	121.0 (8)	N2—C12—C11	122.2 (8)
C2—C3—H3	119.5	N2—C12—H12	118.9
C4—C3—H3	119.5	C11—C12—H12	118.9
C5—C4—C3	115.9 (7)	N2—C13—C14	119.3 (8)
C5—C4—C7	122.6 (7)	N2—C13—H13	120.4
C3—C4—C7	121.5 (8)	C14—C13—H13	120.4
C4—C5—C6	121.5 (7)	C10—C14—C13	122.2 (8)
C4—C5—H5	119.2	C10—C14—H14	118.9
C6—C5—H5	119.2	C13—C14—H14	118.9
N1—C6—C5	120.6 (8)	N2—C15—H15A	109.5
N1—C6—H6	119.7	N2—C15—H15B	109.5
C5—C6—H6	119.7	H15A—C15—H15B	109.5
C4—C7—C8	110.6 (7)	N2—C15—H15C	109.5
C4—C7—H7A	109.5	H15A—C15—H15C	109.5
C8—C7—H7A	109.5	H15B—C15—H15C	109.5

## References

[bb1] Bruker (1998). *SMART* Bruker AXS, Inc., Madison, Wisconsion, USA.

[bb2] Bruker (2003). *SAINT-Plus* Bruker AXS, Inc., Madison, Wisconsion, USA.

[bb3] Dou, Y.-L., Li, Z.-G., Xu, J.-W. & Zhang, W.-X. (2007). *Acta Cryst.* E**63**, o1874–o1875.

[bb4] Sheldrick, G. M. (2003). *SADABS* Bruker AXS, Inc., Madison, Wisconsion, USA.

[bb5] Sheldrick, G. M. (2008). *Acta Cryst.* A**64**, 112–122. 10.1107/S0108767307043930 18156677

[bb6] Yang, F., Deng, J.-C., Li, Z.-G. & Xu, J.-W. (2008). *Acta Cryst.* E**64**, o253. 10.1107/S1600536807064963 PMC2915310 21200818

